# How to Sweep a Room

**Published:** 1875-11

**Authors:** 


How to Sweep a Room.—An uninstructed Bridget, armed with a broom is about as charming an occupant of a parlor, or a library well stocked with the pretty little knicknacks which cultivated people like to have about them, as the celebrated bull in the china shop. Before Bridget's entrance, all fragile movables should be stored by careful hands in some neighboring closet; and the furniture, as far as possible, protected by covers and slight draperies, kept for the purpose. Then, after doors have been closed and windows opened, Bridget may be called in and instructed. Almost hopeless the task may seem at first; but after a little she will learn to spread the moderately damp coffeegrounds and tea leaves, or, still better, the slightly moistened bran, evenly over the floor; to brush the corners of the room, and under and back of the heavy articles of furniture, with a parlor brush; then to take her broom, being careful lest its handle shall prove destructive to mirrors or window glass, and instead of digging into the hapless carpet, wearing off the nap and raising clouds of dust by her short strokes, to take long, smooth, straight strokes, the "right way" of the carpet. This manner of handling the broom, together with plenty of the moist bran, will prevent the whirlwinds of dust which otherwise rise, and, penetrating the best arranged coverings, settle everywhere upon books and furniture.

---

CRYSTALLIZED FLOWERS. — Construct some baskets of fancy form with pliable copper wire, and wrap them with gauze. Into these tie to the bottom violets, ferns, geranium leaves—in fact any flowers except full-blown roses—and sink them in a solution of alum, of one pound to a gallon of water, after the solution has cooled. The colors will then be preserved in their original beauty, and the crystallized alum will hold faster than from a hot solution. When you have a light covering of crystals that completely covers the articles, remove the baskets carefully, and allow to drip for twelve hours. These baskets make a beautiful parlor ornament, and for a long time preserve the freshness of the flowers.

